# Rethinking healthcare’s approach to ACEs treatment and the role of social health programming

**DOI:** 10.1038/s41390-025-03970-w

**Published:** 2025-02-28

**Authors:** Rebecca A. Drakowski, Ann L. Anderson-Berry

**Affiliations:** University of Nebraska Medical Center, Department of Pediatrics, Omaha, NE USA

## Abstract

- This correspondence adds to the existing literature by highlighting the importance of community partnerships and social health programming as a necessary component of medical care for adverse childhood experiences.

## Introduction

As evidenced by increasing rates of obesity, hypertension, diabetes, early cancer diagnosis, and decreasing life expectancy in the United States, our population is plagued by worsening health outcomes from non-communicable disease (NCD).^1,2^ There is robust evidence that the development of NCD is associated with exposure to adverse childhood experiences (ACEs).^3^ Nearly two-thirds of adults in the United States have experienced at least one ACE.^3,4^ There is no debate that certain ACEs are entirely unacceptable for any child to experience such as physical or sexual abuse and neglect. However, other ACEs, such as child hunger, housing instability, poverty, parental separation from mass incarceration, or substance use epidemics, are generally recognized as unfortunate consequences of systemic inequities, but very little progress has been made in eliminating these inequities. Still, other ACEs, such as peer-to-peer bullying, emotional abuse, racial discrimination, and parental divorce have become embedded into American culture and are accepted rather than recognized as the traumatic experiences they truly are.

When children are exposed to ACEs, the trauma they experience does not disappear when they reach adulthood. ACEs continue to impact all aspects of a person’s mental and physical health throughout their life via weathering.^3^ Weathering encompasses the cumulative effect of chronic exposure to ACEs and other psychological stressors, which elevates the biological stress response, disrupts multiple biological systems, and may induce NCDs. However, even among individuals with similar ACEs exposure, the severity of weathering may differ. Individuals with the resiliency skills to overcome trauma and cope with stress often experience milder weathering and ultimately have better health outcomes.^5,6^ Traditional healthcare-adjacent treatments for ACEs attempt to mitigate the repercussion of ACEs through referrals to government or community aid organizations and mental health counseling; however, these services are notoriously underfunded and difficult to access. Preventative solutions are needed as part of a holistic approach to build community and improve both pediatric and adult health outcomes through early treatment for ACEs.

## The Role of Social Health Programming

**Fig. 1:**
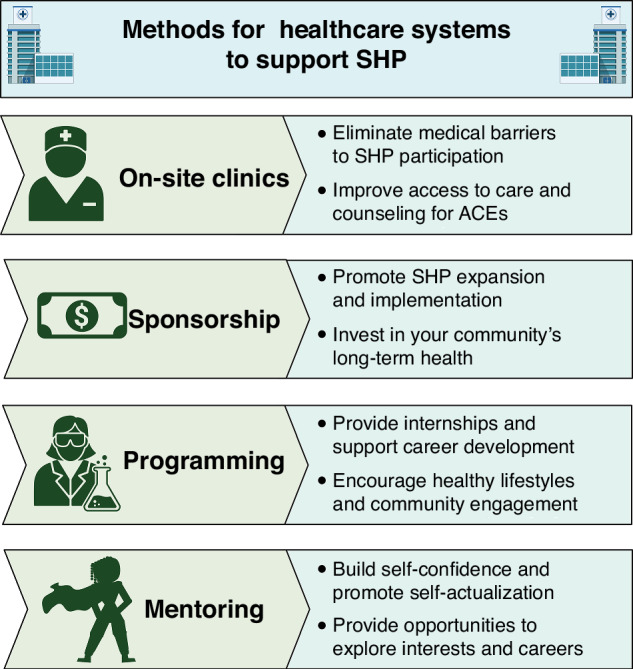
Methods for and benefits of healthcare systems supporting SHP as part of a solution to mitigate the detrimental effects of ACEs. Opportunities for healthcare systems to partner with SHP include the creation of on-site clinics, sponsorship, providing programming, and providing mentoring.

Expanding high-quality after-school and summer social health programming (SHP) is an intervention that can minimize the impact of or even reduce ACEs exposure through multi-modal training and support,^7^ thereby preventing the development of NCDs. SHP for pediatric populations is a childcare model that intentionally addresses ACEs by demanding inclusivity, providing economic opportunities, and empowering families to cope with previous trauma by building resiliency skills. SHP delivered in children’s neighborhoods by local, trusted adults serves to provide mentors and allies, knitting a neighborhood together through support and care for its youngest members. Although SHP is tailored to meet the needs of local communities, hallmarks of SHP include mentorship and counseling availability, tutoring and internship opportunities, culturally tailored and engaging health and nutrition education, and exploration of personal interests such as art, gardening, cooking, or sports. These opportunities have a substantial positive impact on children’s physical, emotional, and mental health.^8,9^ Healthcare systems should partner with local SHP to increase access, provide programming or mentorship, and engage with families in productive conversations about health (Fig. 1).

## Opportunities for Healthcare Systems to Partner with SHP

The creation of on-site clinics at organizations providing SHP is one potential avenue for healthcare systems to alleviate barriers to SHP participation. For example, our academic healthcare system has a collaborative community clinic providing on-site healthcare for local community members and SHP participants at Girls Inc., Omaha. Girls can access the clinic during SHP attendance, and the clinic is open in the evenings to improve access for family or community members who work during typical business hours. The collaborative community clinic can also provide patients with traditional treatment for ACEs, including mental health counseling, referrals to government programs including SNAP (Supplemental Nutritional Assistance Program) or TANF (Temporary Assistance for Needy Families), community resources such as food banks or legal aid, and treatment for NCDs which may have developed because of weathering. Additionally, the longstanding partnership between Girls Inc., Omaha and our healthcare system facilitates the timely delivery and uptake of health information during community health emergencies, such as the COVID-19 pandemic. Partnerships between community organizations delivering SHP and their local health systems are a benefit to all – SHP is improved by reducing barriers to healthcare provision, partnering heath systems are able to build trust in their local communities, and community members receive critical access to both SHP and medical care to address their health needs.

Healthcare systems can also support SHP through sponsorship. Funding is a limiting factor for any organization, and in 2019 the American Academy of Pediatrics released a statement calling for investments in SHP as a solution to mitigate the lifelong biological impact of ACEs.^10^ Sponsorship could support SHP expansion through infrastructure updates, additional staff members, or the implementation of key programming goals. As an example, the University of Nebraska Medical Center (UNMC) partners with multiple SHP entities to host annual science and medicine events where providers and staff volunteer to provide STEM exposure with hands-on STEM activities such as DNA extraction, sheep eye dissection, and neonatal intubation. Additionally, UNMC partners to provide paid internship opportunities for high school students participating in SHPs. These career development opportunities can drastically alter the career trajectory of students who otherwise would not have access to crucial economic, educational, or networking resources. Early investment in SHP as part of an evidence-based preventative medicine strategy also benefits local health systems by promoting workforce pipeline development and potentially reducing later NCD-related healthcare costs.

Individual healthcare providers can support SHP by volunteering to provide educational programming as a representative of their healthcare system. The authors have several years of experience providing programming for SHP both as individuals and as representatives of our healthcare system. SHP programming is an opportunity for healthcare providers to engage with their local community and help children build resiliency skills and healthy lifestyle habits through fun, hands-on activities. Examples of programming we have conducted include nutrition and cooking classes, self-defense seminars, science-themed escape rooms, art projects, and water balloon fights. Additionally, healthcare providers can support the educational and career development of SHP participants by volunteering to provide tutoring, hosting a student intern, or becoming a mentor for one child. Healthcare providers can be particularly impactful mentors for children interested in medical or scientific careers, as they can share their own experiences with their mentees and provide otherwise unattainable opportunities for mentees to explore career options. As a mentor, healthcare providers can also provide invaluable emotional support and help children build their self-confidence. These interactions enrich SHP experiences for participants, are fulfilling and impactful experiences for volunteers, and foster community trust in local health systems.

## Conclusions

The long-term benefits of SHP for both individuals and communities are positive, measurable, and have the potential to improve children’s quality of life and lifespan. In contrast to previous healthcare-based strategies for ACEs treatment – which have historically relied on piecemeal referrals to inconsistent and overburdened government programs like SNAP or TANF – SHP can both reduce ACEs exposure and increase resiliency, thereby mitigating the effects of weathering and potentially preventing unnecessary morbidity and mortality. Healthcare providers must lead the way in advocating for SHP investment both as a preventative treatment for weathering and as part of a holistic approach to addressing health disparities by replacing discriminatory policies with equitable practices.
